# Genetics of sinoatrial node function and heart rate disorders

**DOI:** 10.1242/dmm.050101

**Published:** 2023-05-17

**Authors:** Lieve E. van der Maarel, Alex V. Postma, Vincent M. Christoffels

**Affiliations:** ^1^ Department of Medical Biology, Amsterdam Cardiovascular Sciences, Amsterdam Reproduction and Development, Amsterdam University Medical Centers, University of Amsterdam, Amsterdam 1105 AZ, The Netherlands; ^2^ Department of Human Genetics, Amsterdam Cardiovascular Sciences, Amsterdam Reproduction and Development, Amsterdam University Medical Centers, University of Amsterdam, Amsterdam 1105 AZ, The Netherlands

**Keywords:** Arrhythmia, Genetics, Sinoatrial node

## Abstract

The sinoatrial node (SAN) is the primary pacemaker of the mammalian heart, initiating its electrical activation and ensuring that the heart's functional cardiac output meets physiological demand. SAN dysfunction (SND) can cause complex cardiac arrhythmias that can manifest as severe sinus bradycardia, sinus arrest, chronotropic incompetence and increased susceptibility to atrial fibrillation, among other cardiac conditions. SND has a complex aetiology, with both pre-existing disease and heritable genetic variation predisposing individuals to this pathology. In this Review, we summarize the current understanding of the genetic contributions to SND and the insights that they provide into this disorder's underlying molecular mechanisms. With an improved understanding of these molecular mechanisms, we can improve treatment options for SND patients and develop new therapeutics.

## Teaser

**Summary:** This Review summarizes the development, function and dysfunction of the sinoatrial node, the primary pacemaker of the mammalian heart.

## Introduction

The sinoatrial node (SAN) is a heterogeneous and complex structure in mammalian hearts that is located at the junction of the systemic venous return and the right atrium. The SAN forms part of the cardiac conduction system (Box 1) and initiates the electrical activation of the heart, modulating cardiac function and output to meet physiological demand. Impaired pacemaker function or action potential propagation from the SAN to the atrial myocardium can result in complex cardiac arrhythmias, culminating in sick sinus syndrome or SAN dysfunction (SND) (Wallace et al., 2021; Alpert and Flaker, 1983). Patients with SND can develop sinus bradycardia (see Glossary, Box 2), sinus arrest or block (Box 2), alternating bradyarrhythmia and tachyarrhythmia (Box 2), chronotropic incompetence (Box 2), syncope and increased susceptibility to atrial fibrillation (AF; Box 2) (Box 1) (John and Kumar, 2016). Although advancing age is the strongest risk factor, SND is a complex, multifactorial disease; underlying conditions, such as pre-existing arrhythmias or congenital disorders, and genetic variation can predispose individuals to SND (Peters et al., 2020; Wallace et al., 2021). Inherited and *de novo* pathogenic variants can also contribute to SND, particularly in genes associated with SAN function. However, such pathogenic variants show variable phenotypic effects in human populations, and their role in SND is not fully understood (Wallace et al., 2021). There is no existing therapy that directly addresses the primary underlying causes of SND, and the current clinical management of symptomatic patients is primarily restricted to the (permanent) implantation of electronic pacemakers (Kusumoto et al., 2019).

**Box 1. An introduction to the cardiac conduction system**

The electrical impulse that triggers the coordinated depolarization of atrial and ventricular cardiomyocytes that drives the heart's rhythmic contractions is initiated and distributed throughout the heart by specialized tissues of the cardiac conduction system. The sinoatrial node (SAN), situated at the junction of the systemic venous return and the right atrium, is the primary pacemaker and controls the heart rate. It is a small structure, comprised of only thousands of cells. It houses specialized pacemaker cardiomyocytes that spontaneously generate action potentials and is innervated by the autonomic nervous system that regulates the heart rate, adapting it to meet the cardiac demand. The action potentials generated by pacemaker cardiomyocytes are rapidly propagated from the SAN through the myocardium of the right and left atria, towards the atrioventricular node (AVN). Here, the propagation of the action potentials slows down as the atria contract and the ventricles fill. When the impulse reaches the ventricular conduction system through the AVN, it rapidly propagates into the ventricles through the atrioventricular bundle (AVB), left and right bundle branches and is distributed through the ventricular myocardium by the Purkinje fibre network, triggering the synchronized contraction of the ventricles and the expulsion of blood through the aorta and pulmonary artery.

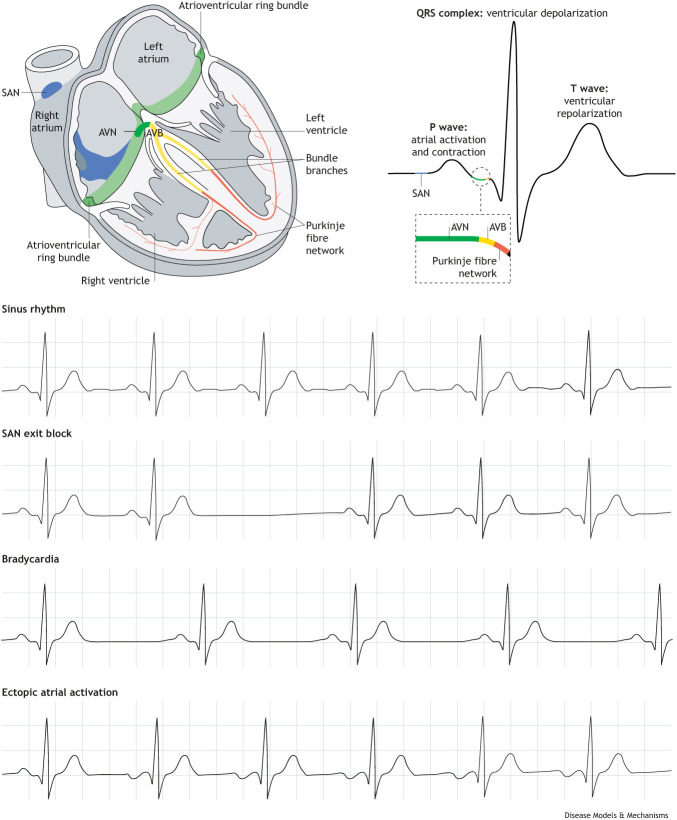

The electrocardiogram (ECG; above) visualizes the propagation of electrical impulses and the collective function of the cardiac conduction system. The P wave arises from atrial activation, initiated by the SAN. The PR interval is the time it takes for the action potential to propagate from the SAN through the AVN and AVB to the ventricles. The QRS, which combines the Q, R and S waves on most ECG traces, visualizes the depolarization of the ventricles while the T wave shows ventricular repolarization.

SAN dysfunction causes abnormal cardiac rhythms, which appear as various waveforms on the ECG. In bradycardia, the P wave is initiated at a slower rate than the healthy 60 times/min. SAN exit block, when the electric impulse generated by the SAN fails to activate the atria, generate a P wave and activate the heart, manifests as the absence of a P wave at a timed interval. ECGs can also reveal atrial activation from an ectopic source, like the AVN or the left atrium, resulting in alternatively shaped P waves (shown as downward waves in the image).

**Box 2. Glossary**

**Atrial ectopic beats:** when the atria contract due to an additional, premature heartbeat that is independent of normal rhythm originating outside the SAN.

**Atrial extrasystoles:** when the atria contract due to an additional, premature heartbeat.

**Atrial fibrillation (AF):** irregular and often very rapid beating of the atria.

**Brugada syndrome:** a genetic disorder causing abnormal electrical activity of the heart, resulting in abnormal heart rhythms and sudden cardiac death.

***Cis*-expression quantitative trait loci (*cis*-eQTL):** genetic variants that influence the expression of one or more genes.

**Chronotropic incompetence:** inability to increase heart rate adequately during exercise to meet the body’s demand.

**Catecholaminergic polymorphic ventricular tachycardia (CPVT):** inherited cardiac arrhythmia characterized by episodic syncope due to the onset of ventricular tachycardia.

**Linkage disequilibrium:** the non-random association of alleles at different loci in a population.

**Long QT syndrome (LQTS):** cardiac arrhythmia that arises due to an abnormally long QT interval.

**Paroxysmal AF:** AF that stops spontaneously within 7 days of onset.

**PR interval:** the duration of time between the onset of the P wave (atrial activation) and the start of the QRS waves (ventricular depolarization).

**QRS duration:** the time it takes for a stimulus to spread through the ventricles (ventricular depolarization).

**SAN exit block:** when the electric impulse initiated by the SAN is inhibited or blocked before it leaves the SAN and activates the atria. This results in the absence of a P wave on an electrocardiogram.

**Sinus arrest/block:** when the SAN ceases to activate the atria for ≥2 s.

**Sinus bradycardia:** resting heart rate initiating from the SAN at a rate of 60 beats/min or less.

**Single**-**nucleotide polymorphism (SNP):** a germline single-nucleotide substitution at a specific location in the genome present in ≥1% of the population.

**Sinus venosus:** the cardiac compartment that precedes the atrium at the venous pole.

**Tachyarrhythmia:** an abnormal heart rhythm with a ventricular rate greater than 100 beats/min.

**Topologically associating domain (TAD):** a highly interacting genomic region in which DNA interacts more frequently with sequences within the TAD than outside the TAD.

Most data regarding the development and function of the SAN and the aetiology of SND are derived from animal models (van Eif et al., 2018), and primary data on human SAN development are limited (Csepe et al., 2016). Although zebrafish have a relatively simple two-chambered heart, their cardiomyocytes share many electrophysiological properties with their mammalian counterparts, and the molecular mechanisms that govern heart development are conserved (Vornanen and Hassinen, 2016; Ravens, 2018; Martin and Waxman, 2021). Mouse models are commonly used to study the genetic mechanisms of SAN development and function, enabling the recapitulation of patient phenotypes *in vivo* (van Eif et al., 2019). However, although the development, function and overall anatomy of the mouse heart are similar to those of the human heart, mouse and human heart differ significantly in size and heart rate (Huang, 2017; Joukar, 2021; van Eif et al., 2018). The study of the human SAN has, until recently, been limited to *ex vivo* systems (Csepe et al., 2016). Recently, the differentiation of human induced pluripotent stem cells to SAN cardiomyocytes has been found to faithfully recapitulate developmental programmes, allowing for the *in vitro* interrogation of SAN cardiomyocytes during human development (Protze et al., 2017; van Eif et al., 2020; Wiesinger et al., 2022). These tools, combined, have built the foundation of our understanding of the genetics and underlying mechanisms of SND.

In this Review, we summarize our current understanding of how rare pathogenic variants and common genetic variation influence SAN function, heart rate control and the clinical presentation of SND. Because rare pathogenic variants in coding regions of the genome have recently been reviewed elsewhere (Wallace et al., 2021), we will discuss newly identified rare pathogenic variants to illustrate underlying mechanisms of the disease. In addition, we will provide current insights into the mechanisms underlying the effects of common variants associated with heart rate-related traits, heart rate itself and rhythm control.

## SAN development, structure and function

The cardiac conduction system is a network of specialized tissues that initiate the depolarizing current and distribute it to the atrial cardiomyocytes. It then propagates the depolarizing current to the atrial and ventricular cardiomyocytes, driving the heart's rhythmic contractions (Box 1). Specialized pacemaker cardiomyocytes in the SAN spontaneously oscillate their membrane potential, generating rhythmic action potentials that are propagated downstream, stimulating the activation of the entire heart (Box 1).

**Fig. 1.**
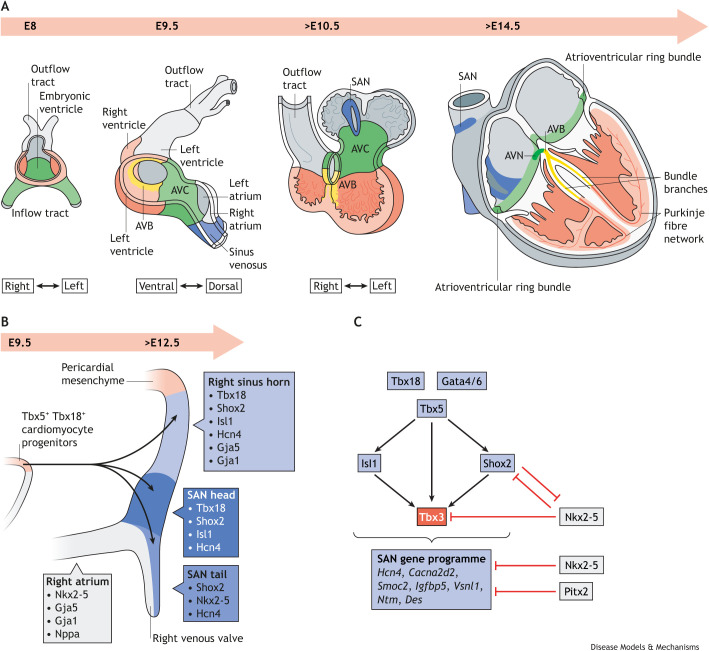
**Development of the cardiac conduction system and the SAN.** (A) On murine E8, the early heart tube has formed and features an embryonic ventricle, outflow and inflow tracts. The heart tube continues to expand from E9.5 onwards, with the addition of cells at the venous pole forming the sinus venosus (blue) while the AVC precursors (green) develop. The SAN develops in the right sinus venosus while the myocardium of the left and right atria and ventricles continues to expand. The AVB develops in the crest of the interventricular septum at the position of the interventricular ring (yellow). The ventricular conduction system takes form, with the right and left bundle branches forming within the ventricular septal trabeculations and the Purkinje fibre network developing from the ventricular chamber myocardium. (B) Development of the SAN: at E9.5, Tbx5^+^/Tbx18^+^ cardiomyocyte progenitor cells from the pericardial mesenchyme will form the sinus venosus and the SAN, and will, from E12.5 onwards, give rise to regions of distinct pacemaker gene expression programmes, including those found in the SAN head and tail. (C) Transcription factor networks direct the activation of the pacemaker gene programme and the atrial working myocardial gene programme in the developing heart. Here, we show how the transcription factors Tbx5 and Tbx3 are at the centre of this network. AVB, atrioventricular bundle; AVC, atrioventricular canal; *Cacna2d2*, Ca^2+^ voltage-gated channel auxilliary subunit alpha2delta2; *Des*, desmin; E, embryonic day; Gata4/6, GATA binding protein 4/6; Gja1/5, gap junction protein alpha 1/5; Hcn4, hyperpolarization-activated cyclic nucleotide-gated K^+^ channel; *Igfbp5*, insulin-like growth factor binding protein 5; Isl1, islet-1; Nkx2-5, NKX2 homeobox 5; Nppa, natriuretic peptide A; *Ntm*, neurotrimin; Pitx2, paired-like homeodomain transcription factor 2, pituitary homeobox 2; SAN, sinoatrial node; Shox2, short stature homeobox 2; *Smoc2*, SPARC-related modular calcium binding 2; Tbx3/5/18, T-box transcription factor 3/5/18; *Vsnl1*, visinin-like 1.

The development of the cardiac conduction system, including the SAN, has been reviewed extensively (van Eif et al., 2018; Bhattacharyya and Munshi, 2020; Park and Fishman, 2017). In brief, spontaneous, asynchronous Ca^2+^ oscillations can already be observed upon formation of the cardiac crescent in the cardiogenic mesoderm by embryonic day (E)7.75 in mouse (Tyser et al., 2016). The first pacemaking centres are formed at the inflow tract of the primary heart tube by E8, initiating slow peristaltic contractions of the primary heart tube (Fig. 1A) (Tyser et al., 2016). However, these centres are not progenitors of the definitive SAN. The initially formed inflow tract myocardium of the primary heart tube is fated to contribute to the atria, whereas the sinus venosus (Box 2) will be added from Tbx18-expressing progenitor cells (Christoffels et al., 2010). This coincides with a shift in dominant pacemaker activity to the newly differentiated cardiomyocytes of the sinus venosus (Fig. 1A) (Christoffels et al., 2010, 2006; Mommersteeg et al., 2010). During looping morphogenesis, specific regions rapidly proliferate and differentiate into chamber myocardium (Fig. 1A) (Moorman and Christoffels, 2003). The chamber myocardial differentiation programmes are suppressed in adjacent regions, including the developing sinus venosus and atrioventricular canal, maintaining the characteristic ‘nodal’ low proliferation rates, slow conduction and automaticity. The SAN will become the dominant pacemaking centre of the E12.5 mouse heart, while the rest of the sinus venosus acquires an atrial myocardial genetic programme (Fig. 1A) (Mommersteeg et al., 2007a; Christoffels et al., 2010; Yi et al., 2012).

*Hcn4*, encoding the hyperpolarization-activated cyclic nucleotide-gated K^+^ channel HCN4, has become a useful functional marker for pacemaker cells in the heart. It mediates the spontaneous activation of pacemaker cells. *Hcn4* expression in the embryonic inflow tract is essential to generate pacemaker potentials; its inactivation in mouse causes severely reduced contraction rates and embryonic lethality between E9.5 and E11.5 (Stieber et al., 2003). *Hcn4* expression is initiated in the cardiac crescent. During elongation of the heart tube, *Hcn4* expression is initiated in the newly added cardiomyocytes at the caudal pole and downregulated in cranial cardiomyocytes. Its expression domain is thus confined to the sinus venosus formed from Tbx18-expressing progenitors between E9.5 and E12. During the murine foetal period, *Hcn4* expression is further confined to the definitive SAN within the sinus venosus, and it remains one of the most specific pacemaker cell markers throughout life (Mommersteeg et al., 2007a; Yi et al., 2012; Liang et al., 2013; Vicente-Steijn et al., 2017). In addition, *Hcn4* is upregulated in the other components of the conduction system during the foetal period.

SAN development is orchestrated by a network of transcription factors that control pacemaker cell differentiation and that is conserved across vertebrates (Fig. 1B,C) (van Eif et al., 2018, 2019; Bhattacharyya and Munshi, 2020; Mandla et al., 2021). Mouse SAN formation is initiated during cardiogenesis when progenitor cells that express the transcription factor genes *Tbx5*, *Tbx18* and *Isl1* form the sinus venosus myocardium. Tbx5 activates the expression of the key pacemaker transcription factor genes *Shox2* and *Tbx3*. These transcription factors, together with Isl1, are the main drivers of the pacemaker gene programme (Fig. 1C) (van Eif et al., 2018; Bhattacharyya and Munshi, 2020; Mandla et al., 2021). Shox2 blocks the initiation of expression of the transcription factor gene *Nkx2-5*, thus maintaining SAN fate and preventing the atrialization of the SAN domain (Hoogaars et al., 2007; Mommersteeg et al., 2007a; Hoffmann et al., 2019; Espinoza-Lewis et al., 2009; Blaschke et al., 2007).

While Nkx2-5 prevents the SAN programme from being activated in the atrial myocardium (Mommersteeg et al., 2007a), the c-isoform of the transcription factor Pitx2 prevents left-sided SAN formation and restricts the developing SAN to the right; *Pitx2* is expressed specifically in the left atrium and left sinus venosus (Mommersteeg et al., 2007b; Wang et al., 2010). At the border of the right-sided atrial myocardium and the SAN, a subpopulation of transitional pacemaker cells that surround the core SAN domain initiate the expression of *Nkx2-5* and of atrial genes, including the gap junction protein-encoding *Gja5* and the Na^+^ voltage-gated channel alpha subunit gene *Scn5a*, which are essential for heart conduction (Hoffmann et al., 2019; Li et al., 2019; Verheijck et al., 1998).

**Fig. 2.**
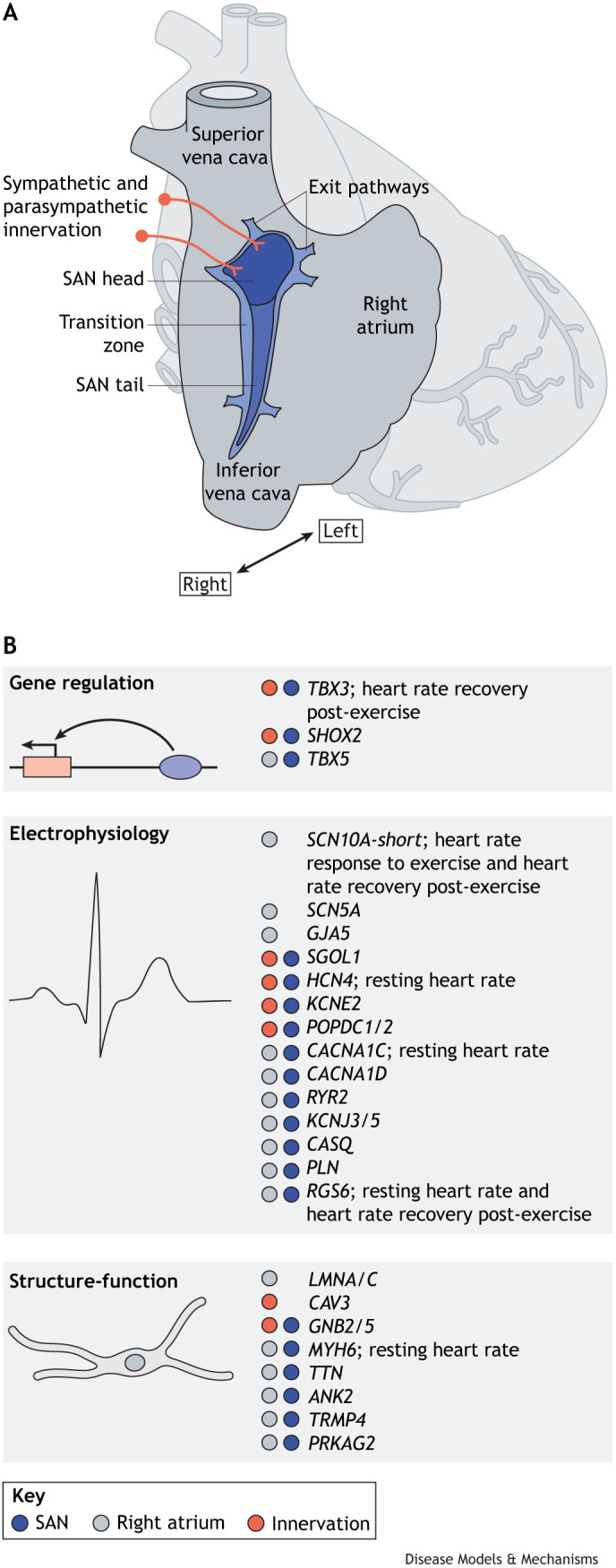
**Genetics of SAN (dys)function.** (A) Structure and anatomical location of the adult human SAN. (B) The expression pattern of pathogenic variants implicated in SND (Table 1) in the right atrium. Several of these candidate variants have also been identified in heart rate-associated GWAS (Eppinga et al., 2016; Nolte et al., 2017; Ramirez et al., 2018; Verweij et al., 2018). *ANK2*, ankyrin-B; *CACNA1C/D*, L-type Ca^2+^ channel subunit Cav1.2/1.3; *CASQ*, calsequestrin-2; *CAV3*, caveolin-3; *GJA5*, gap junction protein alpha 5; *GNB2/5*, guanine nucleotide-binding protein subunit beta-2/5; GWAS, genome-wide association studies; *HCN4*, hyperpolarization-activated cyclic nucleotide-gated K^+^ channel; *KCNE2*, K^+^ voltage-gated channel subfamily E regulatory subunit 2; *KCNJ3/5*, G protein-activated inward rectifier K^+^ channel 3/5; *LMNA/C*, lamin A/C; *MYH6*, myosin heavy chain 6; *PLN*, phospholamban; *POPDC1/2*, popeye domain-containing 1/2; *PRKAG2*, protein kinase AMP-activated non-catalytic subunit gamma 2; *RGS6*, regulator of G protein signaling 6; *RYR2*, ryanodine receptor 2; SAN, sinoatrial node; *SCN5A*, Na^+^ voltage-gated channel alpha subunit 5; *SCN10A-short*, Na^+^ voltage-gated channel alpha subunit 10-short; *SHOX2*, short stature homeobox 2; SND, SAN dysfunction; *SGOL1*, shugoshin 1; *TBX3/5*, T-box transcription factor 3/5; *TRMP4*, transient receptor potential cation channel subfamily M member 4; *TTN*, titin.

The structure and composition of the mammalian SAN have been well characterized (see Easterling et al., 2021; Kalyanasundaram et al., 2023 for recent reviews). In mammals, the SAN has an elongated structure that consists of two domains: the superior ‘head’ that wraps around the superior caval vein at the entrance of the right atrium and the inferior ‘tail’ that extends into the right atrium (Fig. 1B and Fig. 2B) (Wiese et al., 2009; van Eif et al., 2019). The SAN is composed of thousands of specialized pacemaker cardiomyocytes that autonomously oscillate their membrane potential, generating the rhythmic action potentials that activate the atrial myocardium. The SAN is also populated by fibroblasts, endothelial cells, resident macrophages and other cells, which are embedded in the extracellular matrix and contribute to its functionality (Kalyanasundaram et al., 2021; Grainger et al., 2021; Mitrofanova et al., 2018; MacDonald et al., 2020). The mammalian SAN is equipped with fail-safes, such as distributed intranodal pacemakers and conduction pathways, which ensure that it functions even in adverse conditions (Li et al., 2017). In addition, the SAN is extensively innervated by the autonomic nervous system, which modulates heart rate via sympathetic and parasympathetic stimulation to meet physiological demands (Rajendran et al., 2019; Choi et al., 2022). The compositional heterogeneity of the SAN has been further highlighted in recent transcriptomic and proteomic analyses (Vedantham et al., 2015; van Eif et al., 2019; Goodyer et al., 2019; Linscheid et al., 2019; Liang et al., 2021; Qu et al., 2023) that identified the presence of different subpopulations of pacemaker cardiomyocytes in the SAN that share a core pacemaker gene programme. These subpopulations were characterized according to their distinct transcriptional profiles, and include ‘head’ pacemaker cells, ‘tail’ pacemaker cells and so-called transitional pacemaker cells, which have a transcriptional and electrophysiological phenotype in-between that of pacemaker and atrial cardiomyocytes.

**Table 1.**
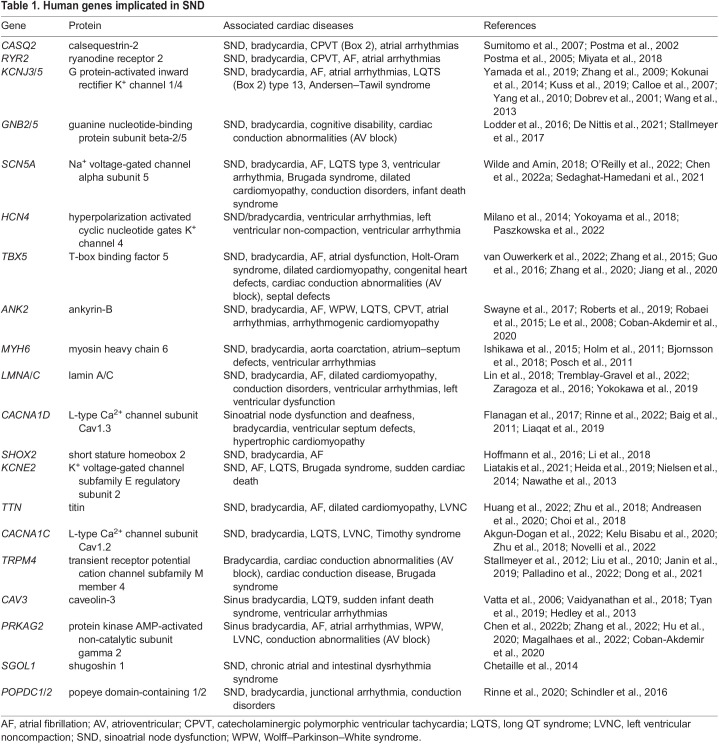
Human genes implicated in SND

SAN function is driven by pacemaker cardiomyocytes and is modulated by the autonomic nervous system and signalling molecules. Pacemaker cell automaticity and rhythm are the products of a strictly regulated internal-coupled clock system that is modulated by the autonomic nervous system. The internal coupled-clock system consists of the Ca^2+^ clock, which is the local diastolic intracellular Ca^2+^ released from the ryanodine receptors (RYRs) of the sarcoplasmic reticulum, and the membrane clock, which consists of plasmalemmal ion channels (including HCN), voltage-gated Ca^2+^ channels and voltage-dependent tetrodotoxin-sensitive Na^+^ channels (Lang and Glukhov, 2018). Although voltage-gated Na^+^ channels have been reported in the SAN of multiple species, recent *ex vivo* studies indicate that these channels may be more involved in SAN conduction rather than automaticity in the human SAN (Li et al., 2020; Verkerk et al., 2009).

The sequential and cyclical activation of the membrane clock and the spontaneous Ca^2+^ clock work together and synchronously ensure pacemaker cell automaticity and the generation of a stable heart rate (Lyashkov et al., 2018; Tsutsui et al., 2021; Vinogradova et al., 2010). The autonomic nervous system regulates the balance between the sympathetic and parasympathetic stimulation of the SAN, controlling pacemaker cell automaticity and SAN function by modulating the membrane and Ca^2+^ clocks (Behar et al., 2016; Vinogradova et al., 2010). This balance is largely maintained through the activation of adenylyl cyclase in pacemaker cells. Sympathetic stimulation via adenylyl cyclase has a positive chronotropic effect by elevating cytosolic cyclic adenosine monophosphate (cAMP) levels in pacemaker cells, accelerating diastolic depolarization, increasing firing rate via HCN4 (Fenske et al., 2020; Tsutsui et al., 2021) and accelerating the onset of local subsarcolemmal Ca^2+^ releases from RYRs (Vinogradova et al., 2010).

Taken together, a network of transcription factors including TBX18, TBX5, TBX3, SHOX2 and ISL1 control the development of the SAN and differentiation of the pacemaker cells (Fig. 1C). Although these specialized pacemaker cardiomyocytes spontaneously generate the electrical impulse that will trigger the heartbeat, they are embedded within a complex, heterogeneous network of cells and extracellular matrix that form the functional SAN. The robust automaticity of pacemaker cells, the modulation of pacemaker cell automaticity by the autonomic nervous system, and the conduction of the electrical impulse to the working myocardium of the right atrium, together, ensure that the heart can keep up with metabolic demand.

## Rare pathogenic variants implicated in SAN dysfunction

Genetic variation, environmental factors and underlying conditions contribute to SND (Jensen et al., 2014; Wallace et al., 2021). Although cases of inherited SND are rare, a few pathogenic variants in coding loci have been implicated in SND (Table 1), revealing their functional importance in normal SAN function and heart rate control. Most cases of inherited SND involve genes that encode ion channels that affect SAN function or structural proteins (see Table 1 and Fig. 2B). Mutations in these genes result in impaired pacemaker cell automaticity or conductance, or in impaired pacemaker cell modulation (Wallace et al., 2021). Perhaps expectedly, several genes encoding the above-discussed cardiac developmental transcription factors that regulate SAN development, including *TBX5*, *TBX3* and *SHOX2*, have also been implicated in SND. Most genes implicated in SND have been reviewed elsewhere (Wallace et al., 2021); here, we discuss some recently identified rare pathological variants that affect SAN gene regulation and function, which have contributed to our understanding of SAN function and dysfunction.

### T-box transcription factor 5 (TBX5)

The T-box transcription factor TBX5 is a key regulator of early mammalian cardiogenesis and of the specification and function of the cardiac conduction system (Steimle and Moskowitz, 2017). This transcription factor plays a key role in regulating the expression of genes that encode ion-handling proteins and regulate heart-contraction rhythm control. The transcriptional targets of TBX5 are involved in cardiac proliferation, maturation and function (ion handling), and include genes that encode ryanodine receptors (e.g. *Ryr2*), Ca^2+^ transporters (e.g. *Atp2a2*), electrolyte regulators (e.g. *Nppa*) and gap junction proteins (e.g. *Gja5*) (Steimle and Moskowitz, 2017). *TBX5* haploinsufficiency causes Holt-Oram syndrome, which is characterized by congenital arm/hand and heart defects and by cardiac conduction system abnormalities (Li et al., 1997; Basson et al., 1997). A pathogenic variant in the 5th exon of *TBX5*, which contains the T-box domain, called p.G125R;c373G>A, was identified in a Dutch family presenting with atypical Holt-Oram syndrome and paroxysmal AF (Box 2) (Postma et al., 2008). A recent analysis of the electrocardiograms (ECGs) of this family's members revealed that they have previously unreported cardiac abnormalities in addition to those described in their medical records, including increased heart rate variability, sinus arrest, atrial ectopic beats (Box 2) and SND (van Ouwerkerk et al., 2022). Mice heterozygous for the patient-derived *Tbx5* p.G125R variant are morphologically normal but are susceptible to AF and present with atrial extrasystoles (Box 2) and SND symptoms, including bradycardia, increased heart rate variability and a prolonged sinus node recovery time (van Ouwerkerk et al., 2022). This Tbx5 variant shows increased and altered interaction with its target regulatory elements, thereby inducing widespread transcriptional and epigenetic changes in both the atrial and SAN cardiomyocytes. The atria of mice heterozygous for *Tbx5* p.G125R differentially express genes involved in Ca^2+^ handling and gap junctions, such as *Cacna1c*, *Pln* and *Gja5*, compared to the atria of wild-type mice. However, the heterozygous mice do not differentially express electrophysiologically relevant genes, such as the aforementioned *Scn5a*, *Ryr2* and *Atp2a2*, that are otherwise known to be affected by *Tbx5* insufficiency (van Ouwerkerk et al., 2022; Zhu et al., 2008; Nadadur et al., 2016). In the right atrial/SAN tissues of *Tbx5* p.G125R heterozygous mice, the expression levels of key pacemaker transcription factor-encoding (*Tbx3*, *Isl1* and *Shox2*) and ion channel-encoding (*Hcn4*, *Hcn1* and *Cacna2d2*) genes, remained unchanged, and their SAN appeared structurally unaffected (van Ouwerkerk et al., 2022). However, several genes involved in pacemaker activity and particularly in Ca^2+^ handling (*Cacna1d*, *Cacna2d3*, *Cacna1g*, *Cacna1h* and *Ryr3*), showed significant differential expression relative to that in wild-type mice, as did genes encoding bone morphogenetic protein-signalling components (*Bmp2*, *Bmp3*, *Bmp10*). Although the precise mechanism by which the TBX5;p.G125R variant causes SND remains to be defined, these findings might shed light on the epigenetic and transcriptional states that predispose to SND.

### Short stature homeobox 2 (SHOX2)

The transcription factor SHOX2 has also been implicated in SND. SHOX2 plays a key regulatory role in murine and human SAN development, driving the activation of the pacemaker gene programme in the developing SAN, and preventing *NKX2-5* expression and the atrialization of the SAN (Espinoza-Lewis et al., 2009, 2011). *Shox2* deficiency causes severe bradycardia in mice (Espinoza-Lewis et al., 2009); mutations in human *SHOX2* have been identified in patients with AF and SND (Li et al., 2018). Of note, AF and SND co-exist, as SND affects one in five patients with AF (Jackson et al., 2017; John and Kumar, 2016). A recent screening for *SHOX2* as a common susceptibility gene for SND and AF led to the identification of a heterozygous missense p.P33R variant in a SND patient cohort. This variant leads to impaired SHOX2 transactivation activity (Hoffmann et al., 2019). SHOX2 mediates the phenotypically intermediate state of transitional pacemaker cells by suppressing the transcriptional output of NKX2-5 through a SHOX2–NKX2-5 antagonistic mechanism (Ye et al., 2015). Mice that lack *Shox2* expression in the SAN junction present with severe SND alongside an absent SAN tail domain (Ye et al., 2015). Moreover, although the inactivation of *Nkx2-5* in the transitional SAN domain specifically does not cause morphological abnormalities, these mice also present with SND (Li et al., 2019). Although the aetiology of SND in patients carrying *SHOX2* variants remains unknown, altered or impaired transcription factor activity can have widespread, detrimental consequences on the epigenetic and transcriptome state of the SAN and right atrium that predispose to SND.

### Popeye domain-containing (POPDC) gene family

The POPDC gene family includes *Popdc1* (also known as *Bves*), *Popdc2* and *Popdc3*. These genes encode transmembrane proteins, and their expression is enriched in the cardiac conduction system and the SAN of higher vertebrates (Froese and Brand, 2008; Alcalay et al., 2013). Morpholino-mediated knockdown of *popdc1* or *popdc2* in zebrafish causes bradycardia (Kirchmaier et al., 2012) (Schindler et al., 2016), while *Popdc1*- and *Popdc2*-null mice present with SND alongside structurally and compositionally modified SAN and morphologically abnormal pacemaker cells (Froese et al., 2012; Unudurthi et al., 2016). Mutations in human *POPDC2* have recently been associated with sinus bradycardia and SND (Gruscheski and Brand, 2021). As the oscillating membrane potential of pacemaker cells is the cumulative product of ion channels, pumps and regulators that could all potentially be influenced by POPDC, the exact electrophysiological mechanisms by which POPDC2 variants cause SND remain unclear. It is likely that SND in these patients is a product of altered POPDC2 interactions with regulators of pacemaker cell automaticity and excitability, including the K^+^ channel TREK1 (also known as KCNK2) (Unudurthi et al., 2016) and CAV3 (Balijepalli and Kamp, 2008; Barbuti et al., 2012). Further investigations of the POPDC variants and their interacting partners will enhance our understanding of the role of altered pacemaker cell automaticity and excitability in SND.

Taken together, rare pathogenic variants in genes that affect SAN gene regulation and function advance our understanding of SAN function and the aetiology of SND. They also illustrate the critical roles of transcription factors like TBX5 and SHOX2 in the development of a functional SAN. Such pathogenic variants also shed light on previously unappreciated downstream genes that directly influence pacemaker cell function, as illustrated by *POPDC2*. However, these human genetic studies have identified only a handful of genes involved in SAN function. Only recently, studies have been initiated to explore the role of common genetic variation in the aetiology of SND.

## Common variants implicated in SAN dysfunction: novel insights from genome-wide association studies (GWAS)

GWAS are a valuable tool for identifying genomic regions associated with traits and complex phenotypes (Cano-Gamez and Trynka, 2020). GWAS help to define the genetic architecture of traits or diseases by identifying common, inter-individual variants in the human genome that include single-nucleotide polymorphisms (SNPs; Box 2) and that usually have small effects on a trait. Although GWAS of SND have, as yet, not been performed, several large GWAS have investigated genetic associations with resting heart rate and its modulation, response to recovery post-exercise, response to exercise and variation (Eppinga et al., 2016; Nolte et al., 2017; Ramirez et al., 2018; Verweij et al., 2018). These traits reflect aspects of SAN function, including intrinsic properties of the SAN itself and autonomic nervous system function, and have a large heritable component, ranging from 10-20% in SNP-based and 30% in family-based analyses to 60% among twins and siblings (van de Vegte et al., 2019; Xhaard et al., 2021). Collectively, GWAS identified genetic variants at >30 independent loci that are significantly associated with one of these traits. As SND and AF co-exist and are inter-related (John and Kumar, 2016), some of the loci identified in the extensive GWAS of AF might inform studies of SAN function, and vice versa. Indeed, although the loci associated with AF and heart rate-related traits differ substantially, several loci have been associated with both AF and heart rate-related traits, including *HCN4*, *SCN10A* and *MYH6* (Ramirez et al., 2018; Roselli et al., 2018; Nielsen et al., 2018). In general, the variants associated with heart rate-related traits are thought to affect the expression or function of the nearest genes considered to be involved in autonomic nervous system and SAN function, thus quantitatively influencing these traits (van de Vegte et al., 2019). However, functional studies of the genetic variant–SAN phenotype relations are still scarce, and the studies discussed below show that variants influence unexpected target genes and mechanisms.

The functional interpretation of GWAS associations, however, is difficult (Cano-Gamez and Trynka, 2020). SNPs associated with disease phenotypes are often found to be in strong linkage disequilibrium (Box 2) with many other common SNPs, meaning that they are inherited together, complicating the identification of the causal variants. Additionally, it is often unclear how potentially causal variants alter genomic function. For example, do they alter the coding sequence, splice sites or regulatory sequences? It is noteworthy that common disease-associated variants are often found in non-coding regions that are enriched for epigenetic signatures associated with gene regulation, including transcription factor-binding sites and sites with increased DNA accessibility, predicting the presence of *cis*-regulatory elements (Maurano et al., 2012; Timpson et al., 2018). Variants that alter regulatory elements by, for example, preventing transcription factor binding or by generating new transcription factor recognition sites, can cause changes in the transcriptional activity and translation of these elements' target genes (Timpson et al., 2018). These changes can compound the effects of other factors, including environmental factors and changes caused by other genetic variants at the same or different loci, thus predisposing to disease. However, it remains unclear how such variant regulatory elements contribute to an associated phenotype, and the genes, cell types and developmental events that they influence have proven difficult to define (Umans et al., 2021). In summary, although no GWAS have been performed for SND, heart rate-focused GWAS reflect SND-relevant characteristics of SAN function, including intrinsic pacemaker properties and autonomic modulation. These studies reveal that there is a large heritable component to these characteristics. Nevertheless, it remains challenging to functionally interpret GWAS and understand how the variant loci they identify alter genomic function.

## GWAS identify common variants in loci implicated in SND

Several genomic loci have been identified in the aforementioned GWAS of traits linked to SAN function. Several of these colocalize with loci that contain genes previously implicated in SND via the characterization of rare pathogenic variants and other developmental and functional studies (Table 1). Notably, candidates such as *MYH6*, *HCN4*, *SCN5A*, *CACNA1C* and *CACNA1D* frequently surface in these studies (Fig. 2B). It is therefore assumed that common variants in these loci contribute to SND predisposition, most likely by influencing the genes' expression level or pattern, or the functions of the encoded proteins. For example, deletion of the mouse orthologue of the human non-coding region that contains common AF-associated SNPs upstream of *HCN4* (Roselli et al., 2018; Nielsen et al., 2018) severely downregulates *Hcn4* in the SAN and alters the expression of the nearby genes, *Loxl1*, *Nptn* and *Neo1* (van Ouwerkerk et al., 2020). Mice homozygous for the deletion of this region die during embryogenesis, similarly to *Hcn4*-deficient embryos (Stieber et al., 2003). Adult mice heterozygous for this deletion present with frequent sinus pauses, reminiscent of previously reported conditional *Hcn4* knockout mice (Herrmann et al., 2007; Harzheim et al., 2008; Hoesl et al., 2008), alongside increased heart rate variability, fragmented QRS (Box 1) and prolonged SAN recovery time after pacing (van Ouwerkerk et al., 2020). These examples indicate that common variants might affect enhancer function and expression levels of enhancer target genes important for SAN function (van Ouwerkerk et al., 2020).

## Variant regulatory elements, target genes and SAN function

As mentioned above, trait-associated variation may modify regulatory DNA sequences that determine tissue- and context-dependent gene expression (Tak and Farnham, 2015; Maurano et al., 2012; Timpson et al., 2018). Genome-wide mapping of chromatin accessibility, which is indicative of active regulatory elements, including enhancers, has been used to identify potential regulatory elements in human and mouse pacemaker cells (van Eif et al., 2020; Galang et al., 2020). These studies have functionally validated enhancers that are essential for the expression of key transcription factor-encoding genes in the SAN, such as *Tbx3*, *Isl1* and *Shox2*. They have also identified and located multiple SNPs associated with resting heart rate near the mouse orthologous region of the conserved *ISL1* enhancer, indicating that this enhancer plays a role in regulation of the human heart rate (Galang et al., 2020).

**Fig. 3.**
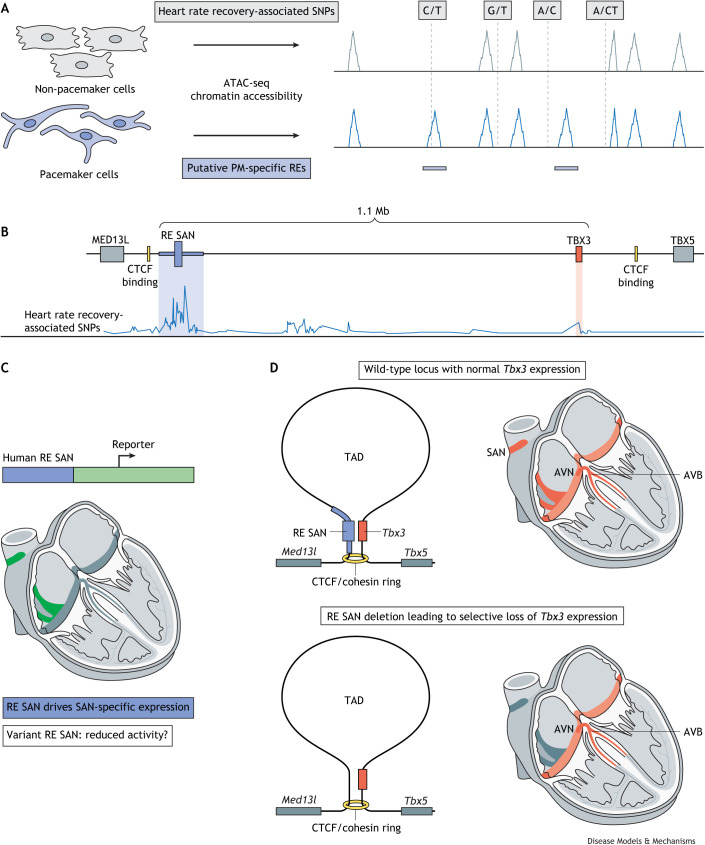
**Genome-wide mapping of SAN-specific regulatory elements as an approach to identify heart rate-associated variant enhancers.** (A) The combination of genome-wide mapping of chromatin accessibility (for example, ATAC-seq) with heart rate-associated SNPs has been used to identify putative pacemaker-specific REs. These analyses can reveal the gene and transcription factor networks that define pacemaker cells (see Fig. 1C). (B) For example, a recent study from our own laboratory (van Eif et al., 2020) used the approach outlined in A to identify several pacemaker cell-specific accessible genomic regions close to the *MED13L* gene. *TBX3* is located in a TAD between the *MED13L* and *TBX5* loci, flanked by CTCF binding sites and spanning a >1 million base pairs (Mb) ‘gene desert’. In this work, we also showed that these pacemaker cell-specific accessible genomic regions can harbour variants that affect heart rate recovery after exercise. Therefore, we have identified a putative genetic mechanism underlying a form of SAN dysfunction. (C) Several putative REs SAN drive gene expression in the developing mouse heart. Researchers can investigate this regulatory mechanism in mouse models by fusing reporter genes to wild-type or variant REs SAN and analysing the expression patterns of the reporters. To validate the translational implications of this approach, researchers can substitute the endogenous murine locus with the human RE SAN (either wild-type or those that carry SNPs) to study how specific genetic variants within regulatory regions affect SAN function and, therefore, to understand the functional role of SND-related genetic variants. SNPs and other variants in the human orthologous RE SAN may influence heart rate-associated traits, including heart rate recovery after exercise, by reducing RE activity and, consequently, *TBX3* expression in the SAN. (D) Deleting the heart rate recovery-related RE (RE SAN) abrogates the expression of *Tbx3*. Murine embryos with a RE SAN homozygous deletion display selective loss of Tbx3 expression from the SAN and (cardiac) ganglia, although Tbx3 expression is maintained in the embryonic atrioventricular conduction system and other tissues, such as lungs and liver (these are not shown). However, such homozygous deletion is embryonic lethal. Heterozygous animals survive to adulthood but show prolonged SAN recovery times after exercise, which indicates that correct Tbx3 expression levels are not merely required for SAN development, but are also essential for normal SAN function in adulthood (van Eif et al., 2020). ATAC-seq, assay for transposase-accessible chromatin with sequencing; AVB, atrioventricular bundle; AVN, atrioventricular node; CTCF, CCCTC-binding factor; MED13L, Mediator complex subunit 13L; PM, pacemaker; RE, regulatory element; SAN, sinoatrial node; SND, SAN dysfunction; SNP, single-nucleotide polymorphism; TAD, topologically associating domain; TBX3/5, T-box transcription factor 3/5.

GWAS have identified genetic variants associated with heart rate response after exercise that map close to *MED13L*, the presumed causal gene (Verweij et al., 2018). Our group used assay for transposase-accessible chromatin with sequencing (ATAC-seq) to map putative human pacemaker-specific regulatory elements, including enhancers, in pacemaker cells derived from human induced pluripotent stem cells (van Eif et al., 2020) (Fig. 3A), which identified several pacemaker-specific accessible sites (putative regulatory elements) close to *MED13L* (Fig. 3B). Of note, *MED13L*-proximal SNPs associated with heart rate response after exercise colocalize with these putative regulatory elements. Importantly, these human elements were able to drive reporter gene expression specifically in the developing SAN of transgenic mouse embryos (van Eif et al., 2018) (Fig. 3C). *TBX3* is located in a CCCTC-binding factor (CTCF) site-flanked topologically associating domain (TAD; Box 2) between *MED13L* and *TBX5* that covers a >1 million base pairs (Mb) gene desert and includes the SNPs and SAN-specific regulatory elements (Fig. 3B) (van Weerd et al., 2014; van Eif et al., 2018). Regulatory elements and their target genes are usually confined to the same TAD (Panigrahi and O'Malley, 2021; Furlong and Levine, 2018; de Laat and Duboule, 2013). Indeed, the homozygous deletion of the mouse orthologue of this *TBX3*-distal region containing the regulatory elements and heart rate response after exercise-associated SNPs abolished *Tbx3* expression specifically in the SAN, whereas its expression was maintained in most other tissues, including the atrioventricular conduction system (Fig. 3D). Importantly, the expression of *Tbx5* and *Med13l* was not affected by the deletion of this regulatory element.

Mice homozygous for the deletion of the *Tbx3*-distal region die around birth, much later than *Tbx3*-deficient mice (Davenport et al., 2003; Bakker et al., 2008; Frank et al., 2011). Consistent with the effect of heart rate response after exercise-associated variants in humans, adult mice heterozygous for this deletion, which reduces *Tbx3* expression in the SAN, had a lower heart rate, increased heart rate variation and prolonged SAN recovery time after pacing. *Tbx3* expression in these mice was also reduced in the neurons that innervate the SAN. Although blocking the function of these neurons did not rescue normal resting heart rate or SAN recovery time after pacing, it did alleviate the increased heart rate variation. This study strongly suggests that the heart rate response after exercise-associated variants close to *MED13L* affect the functioning of regulatory elements that are necessary for *TBX3* expression in the SAN and autonomic nervous system, thereby affecting the function of these tissues and heart rate response after exercise.

## Common *SCN10A* variants modulate transcription of a novel *SCN10A* isoform

GWAS unexpectedly identified genetic variants in *SCN10A* that were strongly associated with AF, Brugada syndrome (Box 2), cardiac conduction velocity, heart rate, and heart rate response to exercise and after exercise (Bezzina et al., 2013; Roselli et al., 2018; Nielsen et al., 2018; Eppinga et al., 2016; Nolte et al., 2017; Ramirez et al., 2018; Verweij et al., 2018). *SCN10A* encodes the alpha subunit of the neuronal voltage-gated Na^+^ channel Nav1.8, but its cardiac function has yet to be fully resolved (Hu et al., 2014; van den Boogaard et al., 2014). One hypothesis that seeks to explain the association is that *SCN10A* SNPs influence the function of an important enhancer in an intron that controls the cardiac expression of the adjacent *SCN5A* (van den Boogaard et al., 2012, 2014). *SCN5A* encodes the alpha subunit of the major cardiac Na^+^ channel, and is essential for impulse conduction and heart function. *SCN5A* mutations have previously been implicated in arrhythmia disorders such as Brugada syndrome and in SND (Table 1) (Wallace et al., 2021). These *SCN5A* mutations that hamper activation and inactivation of the inward Na^+^ current compromise pacing in peripheral, but not in central, pacemaker cells and induce SAN exit block (Box 1, Box 2) by limiting action potential propagation across the SAN–atrium interface *in vitro* (Butters et al., 2010). *Scn5a^+/−^* mice, too, present with compromised pacing in peripheral pacemaker cells and exhibit SAN exit block (Lei et al., 2005).

A recent study reported the expression of a mouse *Scn10a* transcript that comprises the last seven exons of *Scn10a*, called *Scn10a-short*, that encodes Nav1.8-short. This transcript is expressed in the atria, SAN and ventricular conduction system of mice, whereas full-length *Scn10a* transcripts are undetectable in the human and mouse heart (Man et al., 2021). The transcription of *Scn10a-short* initiates from the conserved intronic enhancer–promoter complex in *Scn10a/SCN10A* mentioned above. A common SNP, rs6801957 (Man et al., 2021), is associated with arrhythmias like Brugada syndrome and AF, and with electrophysiological traits such as PR interval (Box 2) and QRS duration (Box 2) (Bezzina et al., 2013; van Setten et al., 2018; Ntalla et al., 2020; Sotoodehnia et al., 2010). Two other SNPs, rs6599250 and rs6795970, which are also associated with arrhythmia and *SCN10A* function (van Setten et al., 2018; Ntalla et al., 2020; Sotoodehnia et al., 2010), are in linkage disequilibrium with rs6801957. The SNPs in this haplotype show a significant *cis*-expression quantitative trait loci (*cis*-eQTL; Box 2) effect with *SCN10A* in cardiac tissue, indicating that they significantly affect *SCN10A* expression in *cis*. The cardiac expression of *Scn10a-short* was abolished in mice that carry a homozygous disruption of the *Scn10a* intronic enhancer, while, surprisingly, their *Scn5a* expression was barely affected (Man et al., 2021). Mice with a disrupted enhancer exhibit slowing atrial conduction, atrial arrhythmia and SAN exit block, and their atrial cardiomyocytes show a strongly reduced cardiac Na^+^ current. Cell culture experiments revealed that Nav1.8-short (encoded by *SCN10A-short*) itself is not functional but it enhances the Nav1.5-driven Na^+^ current (Man et al., 2021). Taken together, these studies identify a causal link between arrhythmia-associated SNPs, modulation of tissue-specific target gene expression, and heart rhythm modulation.

## Conclusions and perspectives

The genetic contributions to SND are complex and multifactorial. Although rare familial mutations emphasize key drivers of robust SAN function, GWAS have revealed that common genomic variation in the human population substantially influences heart rate-related traits. These are relevant indicators of SAN function, both its intrinsic and autonomic modulation, and SND risk, revealing a substantial underlying genetic component. Recent efforts in SAN/SND research have been focused on three main goals: (1) identification of targets for treatment, (2) regeneration of the dysfunctional SAN, or (3) replacement of a dysfunctional SAN with an engineered biological construct (Mesirca et al., 2021; Cingolani et al., 2018; Boink et al., 2015). To reach these goals, we require a deeper understanding of the molecular and cellular complexity of SAN function and its interaction with the autonomic nervous system.

The generation of biological pacemakers, either by programming cells in the heart by gene therapy or by developing functional pacemaker cells or tissues *in vitro* for transplantation, requires an understanding of the genetic and epigenetic processes of SAN development. For example, TBX18 was identified as a key transcription factor for SAN development (Wiese et al., 2009) and was subsequently found to be able to generate pacemaker activity in postnatal ventricular tissue *in vivo* (Cingolani et al., 2018). In recent efforts, insights from developmental biology were used to differentiate functionally relevant pacemaker cell subtypes from human induced pluripotent stem cells *in vitro*, including central (head), peripheral (tail) and transitional pacemaker cells (Wiesinger et al., 2022). Such efforts serve as important steps towards refining engineered biological pacemakers.

Uncovering the functional consequences of pathogenic mutations in coding regions is relatively straightforward, as they can be readily studied in relevant model organisms. Such studies have indeed provided insights into the complexity of SAN function and have yielded sometimes unanticipated mechanisms that can be useful starting points for the prediction of treatment targets. For example, SND in mouse models lacking HCN channel activity or L-type Cav1.3 Ca^2+^ channels (*Cacna1d* deficient) was partially rescued by genetic ablation of muscarinic G protein-activated channels via Kcnj5 (Mesirca et al., 2016; Mesirca et al., 2014). Thus, although *HCN4*, *CACNA1D* and *KCNJ5* loss-of-function variants have each individually been implicated in SND, combined loss of two of these genes, for example *Hcn4-Kcnj5* or *Cacna1d-Kcnj5*, seems to normalize SAN function. The findings from these mouse models indicate that targeting KCNJ5 either via gene therapy or pharmacological interventions may be a basis for further management of SND (Mesirca et al., 2016). Further insights into the role of single genes or small gene sets will be gained by using refined genetically modified mouse models and the development of engineered human SAN tissue models that can be readily genetically modified. These may yield additional potential targets to treat SND-related morbidities.

Genetic variation is a much more common source of genetic influence on SAN function. However, it remains a challenge to define how inter-individual variation in regulatory elements can change gene regulatory networks and their functional outputs that define an individual's SAN function, including heart rate itself and its response to physiological triggers. The key outstanding questions are which variants in the noncoding genome influence which genes or genetic components, in which direction (upregulation or downregulation), which cell type (e.g. pacemaker cells, adrenergic nerves, fibroblasts) and under which conditions (age-dependent or pathological transcriptional networks and epigenetic states)? How do all these cell type- and condition-specific differences in regulatory network output, which are mostly small to moderate, interact and define the phenotypes of the different cell types of the SAN? Answers to these questions will uncover modifiable or ‘druggable’ targets for gene therapeutic or pharmacological interventions that can improve SAN function. Additionally, modifiable targets can provide insights into genotype–phenotype relationships, which are essential for assessing individual SND risk along with the underlying pathophysiological scenario. Given the anticipated small effect sizes of heart rate trait-associated variants at a particular locus, the complex and heterogeneous cellular composition of the SAN, the spatio-temporal distribution of its functionalities and its small tissue size, answering these questions will be extremely challenging.

The use of animal models to functionally evaluate risk loci, as discussed in this Review, provides a preview of the complexity of variant-influenced gene regulation and SAN function. In addition, animal models have provided proof of concept that regulatory elements, which are subject to variation, control SAN function through their regulation of sometimes unanticipated mechanisms. Owing to the moderate evolutionary conservation of regulatory elements between human and model organisms, including mouse (Villar et al., 2015), and their intrinsic low-throughput nature, these approaches are not suitable to gain the required detailed information for all associated loci. Future human genetics studies will provide whole-genome sequences of individuals with heart rate- or SND-relevant traits, haplotype-specific sequences, better phenotype characterization, and transcriptomes and epigenomes at cellular resolution. These types of data will allow us to start answering questions regarding the variant–target/genes–target/tissue–SAN functional associations. In addition, the development of engineered SAN tissues derived from patient-specific stem cells that recapitulate relevant aspects of SAN function will provide the tools required to functionally study the mechanisms connecting genetic variants and phenotypic output. These models are potentially suitable for high-throughput testing of variant regulatory elements, for genetic modification, for patient-specific genotype studies and for drug intervention testing (Li et al., 2022). Important candidate targets and mechanisms can subsequently be modelled *in vivo* and tested in the context of the entire organism, with its complexity and multi-layered homeostatic control mechanisms. Going one step further, with the help of artificial intelligence (AI) technology, the collective genomic, molecular, cellular and functional data could be integrated to create computer models of functional SAN tissues, including the intrinsic and extrinsic controlling feedback mechanisms. Such models will improve over time and will enhance predictions of effectiveness of candidate therapeutic interventions, and ultimately may become able to predict genotype–SAN function relations and SND probability.
